# Epidemiology of psychiatric disorders following cytoreductive surgeries plus hyperthermic intraperitoneal chemotherapy: a prospective cohort analysis

**DOI:** 10.1038/s41598-023-42047-8

**Published:** 2023-09-07

**Authors:** Guillaume Economos, Vahan Kepenekian, Cécile Barbaret, Laurent Villeneuve, Julie Haesebaert, Olivier Glehen

**Affiliations:** 1 Hospices Civils de Lyon – Centre de soins palliatifs – Hôpital Lyon Sud, Pierre-Bénite, France; 2 Claude Bernard Université Lyon 1 - EA3738 –Centre pour l’innovation en cancérologie de Lyon, Faculty Lyon Sud, Oullins, France; 3 Hospices Civils de Lyon - Department of Surgical Oncology, Hôpital Lyon Sud, Pierre-Bénite, France; 4 Grenoble University Hospital – Service de soins palliatifs, La Tronche, France; 5 Hospices Civils de Lyon - Unité Recherche et Epidémiologie Cliniques - Pôle de Santé Publique, Hôpital Lyon Sud, Pierre, Bénite, France; 6 Research On Healthcare Performance (RESHAPE), Université Claude Bernard Lyon 1, INSERM U1290, Lyon, France; 7 Hospices Civils de Lyon, Pôle Santé Publique, Service Recherche et Epidémiologie Cliniques, 69003 Lyon, France

**Keywords:** Gastrointestinal cancer, Surgical oncology, Psychology

## Abstract

The peritoneal surface malignancy (PSM) is an advanced disease, the prognosis of which has been radically improved since the development of cytoreductive surgery (CRS) with or without hyperthermic intraperitoneal chemotherapy (HIPEC). These procedures are associated with many complications. However, very few data are available regarding the psychiatric morbidities that might occur. The present study assessed the epidemiology of depressive mood and anxiety during the 6 months following the procedure. The analysis of a prospective cohort that included patients who underwent CRS with or without HIPEC between December 2016 and December 2019 was performed. A total of 115 patients were included. During the 6-months follow-up, the mean (SD) Hospital Anxiety and Depression Scale –D (HADS-D) score was 7.8 (48) and a significant increase compared with the pre-operative period (t(49) = − 4.36, p < 0.005) was found. Thirty-seven patients (32%) had a HADS-D score higher than 7. The incidence of a HADS-D score higher than 7 during the follow-up was 0.05 patient per patient-month. Anxiety and the overall mental disorders intensity scores also increased. The results showed an important increase of mental disorders and their intensity during the 6-months following a CRS with or without HIPEC.

## Introduction

The peritoneal surface malignancy (PSM) is an invasion of the peritoneal serosa due to malignant cells from a heterogeneous group of primary tumors that has a poor prognosis^1^. Despite existing primary peritoneal malignancies, the epidemiology of PSM is mainly due to the metastatic spread of ovarian and colorectal cancers on the peritoneal serosa^1,2^. This condition used to have a poor prognosis, the 10 year survival is lower than 20%, regardless of the primary tumor site^3,4^. In the past decades, its prognosis has been radically improved since the development of cytoreductive surgery (CRS) with or without hyperthermic intraperitoneal chemotherapy (HIPEC)^1^. CRS is a complex surgical intervention that lasts up to 10 h to meticulously excise all macroscopic manifestations of the disease. In addition, to target microscopic manifestations, chemotherapy heated to temperature between 42 and 43 °C can be administered^5^ To achieve a complete resection of the macroscopic disease a series of intricate steps that includes digestive resections, hepatectomies, splenectomies, peritonectomies, and anastomoses is often necessary^1^. Unfortunately, these surgical procedures are prone to a significant morbidity^5,6^, including veno-thrombotic events, infections, anastomotic leaks, fistulas, and complications due to long-term stays in intensive care units^2,5^. In a quarter of the patients, these complications lead to readmission during the 6 months following the procedure, up to 13% require another surgery^7^.

Despite the lack of available evidence, CRS with or without HIPEC could be involved in psychiatric morbidity. It was previously reported that patients with intraperitoneal cancers have a high prevalence of mental health comorbidities; up to 33% of patients with colorectal cancer^8^, and up to 74% of patients with ovarian cancer^9^. Moreover, a psychiatric morbidity has been reported after other gastrointestinal and cancer surgeries, and has the potential to impair the quality of life of patients and lead to poor prognosis^10^.

As mental health disorders are prevalent in this population and the treatments are likely to worsen the patient’s mental health, the primary objective of the present study was to assess the epidemiology of depressive mood in a 6-months follow-up after the CRS with or without HIPEC. The secondary objectives were to assess the epidemiology of anxiety and overall mental disorders and their association with health-related quality of life and surgical complications.

## Methods

### Study design and population

The present study is the analysis of a pre-existing prospective cohort that included all patients who underwent CRS at the Lyon Sud Hospital Center between December 2016 and December 2019. All patients included in the cohort were adult patients (aged 18 years old or older) with a PSM due to ovarian or colorectal primitive location who underwent CRS with or without HIPEC at the Lyon Sud hospital.

### Procedure and outcomes

The evaluation of depression, anxiety and overall mental disorders relied on the scores of the overall Hospital Anxiety and depression Scale (HADS—overall mental disorders), and its subscales, the HADS-D (depressive mood), and HADS-A (anxiety disorders).

The study questionnaires were filled-in by the patients before the surgery and at each visit during their follow-up at the inclusion center.

For the screening of depression and anxiety, we respectively used in the dedicated subscales the cutoffs of 7 and 9 to maximize combined sensitivity and specificity^11^.

The quality of life was assessed using the Global Health Status (GHS) score, extracted from the EORTC-QLQ-C30^12^.

### Analyses

Only patients for whom a HADS-D score was available in the 6 months following the surgery (primary outcome) were included.

Descriptive statistics were expressed as means and standard deviations, and counts and percentages. For dependent variables, Student-t test was used to assess the differences between scores at the time of the inclusion and during the 6-months follow-up. The Chi-square test was used to assess the difference between positive screenings in depression and anxiety at the time of the inclusion and during the 6-month follow-up. The association of HADS scale and subscales scores with the GHS was assessed using the Pearsons’ correlation and the Chi-square test.

All analyses were performed on ISM SPSS Statistics 21 and the differences were considered statistically significant using an alpha risk of 0.05.

### Ethics approval and consent to participate

All patients signed an informed consent to be included in the BIG-RENAPE prospective cohort (ClinicalTrial: NCT02823860). The database obtained all legal authorizations in 2012 and was approved by the institutional ethics committee of the Hospices Civils de Lyon in compliance with the French regulation. The methods were carried out in accordance with the relevant guidelines and regulations.

## Results

Among the 305 patients included in the cohort during the study period, 115 had an available HADS-D score during the 6 months following the surgery and were thus included in the analysis.

The mean (SD) age of patients was 59 (9.4) years, the majority of patients were male (n = 62, 55%); 70% of patients had adenocarcinomas, 22% had mucinous adenocarcinomas, 2% had linitis, 1% had cribliform adenocarcinomas, and 6% of the histology. The mean (SD) peritoneal carcinomatosis index at surgery was 6.9 (5.8) and the mean (SD) duration of surgery was 343.5 (104.5) minutes (Table 1).

**Table 1:** Sample characteristics (the indicated sample size is the sample of patients for whom the information was available).

Characteristic	Mean (SD) or n (%)
Age at diagnosis (years)	59 (SD = 9.4)
Sex (birth-assigned)	
Females	51 (45%)
Males	62 (55%)
Age at surgery (years)	60 (SD = 9.4)
Anatomopathology (OMS 4th edition 2010) (n = 115):	
Adenocarcinoma	80 (70%)
Mucinous adenocarcinoma	25 (22%)
Linitis	2 (2%)
Cribliform adenocarcinoma	1 (1%)
Other types	7 (6%)
Peritoneal carcinomatiosis index (n = 98)	6.9 (SD = 5.8)
Length of the surgery (minutes)	343.5 (SD = 104.5)
Grade III and IV postoperative complications (n = 115)	51 (44%)

### Primary outcome

The mean (SD) HADS-D Score during the 6-months follow-up was 7.8 (4.8) and 37 patients (32%) had an HADS-D score higher than 7. The incidence of HADS-D score higher than 7 during the 6-months follow-up was 0.05 patients per patient-month.

### Secondary outcomes (Table 2)

**Table 2:** Differences in depression, anxiety, quality of life and overall psychiatric disorders using HADS scale at month 6 compared to the pre-operative period.

	Mean pre-operative scores (SD)	Mean during a 6 month follow-up (SD)	Statistics
HADS	5.6 (7.8)	13.2 (7.5)	t(112) = − 9.3, p < 0.005
HADS-D	5.1 (4.5)	7.8 (4.8)	t(49) = − 4.36, p < 0.005
HADS-A	7.6 (3.7)	6.6 (3.7)	t(49) = 2.37, p = 0.024
Global health status	67.1 (23.3)	54.4 (22.0)	t(16) = 2.26, p = 0.038

The mean (SD) HADS score during the 6-months follow-up was 13.3 (7.5), with a significant increase in HADS scores between the assessment at the time of inclusion (mean HADS score = 5.6) and the assessment during the 6-months follow-up (p < 0.05). Similarly, the HADS-D score significantly increased from 5.1 (4.5) at the time of inclusion to 7.8 (4.8) during the 6-months follow-up (p < 0.05). There was no significant difference in HADS-A scores between the time of inclusion and the 6-months follow-up. The mean (SD) GHS score significantly decreased from 67.1 (23) at the time of inclusion compared with 54.4 (22.0) during the 6-months follow-up (p < 0.05).

The number of HADS-D scoring higher than 7 significantly increased between the time of inclusion and during the 6-months follow-up (chi2 = 9.25, (Df = 2) p = 0.01).

The number of HADS-A scoring higher thant 9 significantly increased between the time of inclusion and during the fol6-months follow-up (Yates’ correction applied, chi2 = 7.36, (Df = 2), p = 0.007).

The quality of life measured by the GHS significantly decreased between the time of inclusion and during the 6-months follow-up (t = 2.26, (Df = 16), p = 0.038). There was no statistical correlation between the HADS-D and GHS scores (r =  − 0.28, p = 0.084) nor between the HADS and GHS scores during the 6-months follow-up (r =  − 0.23, p = 0.15).

## Discussion

The present study showed an increase of all scores related to depressive mood, anxiety and mental disorders during the 6-months following CRS in PSM. Theses results highlighted a statistically significant increase of the incidence of depression during the 6-months following a CRS. Although a significant decrease of the health-related quality of life score was found, there was no correlation with mental disorders scores.

The present results highlighted the existence of mental disorders, including depression and anxiety disorders, during the 6 months following a CRS. These results complete those of a recent prospective cohort reported by Oswald et al.^13^; the authors explored the levels of anxiety, depression and stress following CRS with HIPEC until discharge. In this prospective cohort of 169 patients, the authors did not identify increased scores in depression nor stress. However, increased scores of anxiety were reported from surgery to discharge. We do not consider the results of the present study as conflicting with those of this study, as the screening tools and the period of assessment were different between the two studies. However, in both studies, the results reported an increased risk of mental disorders in patients who underwent a CRS. Both studies suffered from a major limitation related to the fact that the cases were not defined using the gold standard^14^.

Major depressive disorders are defined by the fifth version of the Diagnostic and Statistical Manual of Mental Disorders (DSM V) as the presence of at least 5 symptoms, among a list of 9, during the same two week period, in addition to: (i) a significant distress and (ii) the exclusion of any attributable cause, better scientific explanation or history of manic or hypomanic episode. Additionally, as recommended by the DSM V, mental disorders should be diagnosed following a semi-structured clinical interview by a psychiatrist^14^. However, the use of a screening tool is relevant to provide preliminary data regarding the epidemiology of mental health in this population. Additionally, as the depressive disorders are often unrecognized, Gelenberg et al. underlined that using tools to diagnose major depressive disorders is a relevant strategy to improve the diagnosis^15^.

Moreover, due the use of a pre-existing database, the study was restricted to the outcomes collected during the design of the database. For instance, post-traumatic stress disorders that might affect patients following their stay at the intensive care unit following CRS were not collected. Moreover, the results of the present study reported the outcomes of a small cohort without comparative groups, which did not enable to make solid conclusions.

Mental disorders have not been considered in peritoneal cancer surgery research until recently,thus, very few data are available^13^. This might be explained by the lack of evidence regarding the association between mental health and surgical outcomes conversely to nutritional or physical issues that have been extensively studied and led to peri-operative interventions^16^. Overall, in addition to Oswald et al. study, the present results advocate for future research to evaluate the causality and the potential impact of mental disorders following peritoneal cancer surgeries on the patients outcomes. Based on the model of nutrition and physical care in cancer surgery, this might enable the development of mental health interventions to prevent, identify and treat mental disorders in the perioperative period.

## Conclusions

The results showed an important increase of mental disorders and their intensity during the 6-months following a CRS with or without HIPEC. This argues to develop interventions in order to address mental health issues in such settings.

## Data Availability

The datasets analyzed during the current study are available from the corresponding author on reasonable request.
